# Microstructural changes of the white matter in systemic lupus erythematosus patients without neuropsychiatric symptoms: a multi-shell diffusion imaging study

**DOI:** 10.1186/s13075-024-03344-3

**Published:** 2024-05-28

**Authors:** Wenjun Hu, Ziru Qiu, Qin Huang, Yuhao Lin, Jiaying Mo, Linhui Wang, Jingyi Wang, Kan Deng, Yanqiu Feng, Xinyuan Zhang, Xiangliang Tan

**Affiliations:** 1 Department of Medical Imaging Center, Nanfang Hospital, Southern Medical University, Guangzhou, China; 2 School of Biomedical Engineering, Southern Medical University, Guangzhou, China; 3 Guangdong Provincial Key Laboratory of Medical Image Processing and Guangdong Province Engineering Laboratory for Medical Imaging and Diagnostic Technology, Southern Medical University, Guangzhou, China; 4 Department of Rheumatology, Nanfang Hospital, Southern Medical University, Guangzhou, China; 5 Departments of Nuclear Medicine, The First Affiliated Hospital of Sun Yat-Sen University, Guangzhou, China; 6 Philips Healthcare, Guangzhou, China

**Keywords:** Systemic lupus erythematosus, Diffusion kurtosis imaging, Neurite orientation dispersion and density imaging, Tract-based spatial statistics, Atlas-based region-of-interest (ROI) analysis

## Abstract

**Background:**

Diffusion kurtosis imaging (DKI) and neurite orientation dispersion and density imaging (NODDI) provide more comprehensive and informative perspective on microstructural alterations of cerebral white matter (WM) than single-shell diffusion tensor imaging (DTI), especially in the detection of crossing fiber. However, studies on systemic lupus erythematosus patients without neuropsychiatric symptoms (non-NPSLE patients) using multi-shell diffusion imaging remain scarce.

**Methods:**

Totally 49 non-NPSLE patients and 41 age-, sex-, and education-matched healthy controls underwent multi-shell diffusion magnetic resonance imaging. Totally 10 diffusion metrics based on DKI (fractional anisotropy, mean diffusivity, axial diffusivity, radial diffusivity, mean kurtosis, axial kurtosis and radial kurtosis) and NODDI (neurite density index, orientation dispersion index and volume fraction of the isotropic diffusion compartment) were evaluated. Tract-based spatial statistics (TBSS) and atlas-based region-of-interest (ROI) analyses were performed to determine group differences in brain WM microstructure. The associations of multi-shell diffusion metrics with clinical indicators were determined for further investigation.

**Results:**

TBSS analysis revealed reduced FA, AD and RK and increased ODI in the WM of non-NPSLE patients (*P* < 0.05, family-wise error corrected), and ODI showed the best discriminative ability. Atlas-based ROI analysis found increased ODI values in anterior thalamic radiation (ATR), inferior frontal-occipital fasciculus (IFOF), forceps major (F_major), forceps minor (F_minor) and uncinate fasciculus (UF) in non-NPSLE patients, and the right ATR showed the best discriminative ability. ODI in the F_major was positively correlated to C3.

**Conclusion:**

This study suggested that DKI and NODDI metrics can complementarily detect WM abnormalities in non-NPSLE patients and revealed ODI as a more sensitive and specific biomarker than DKI, guiding further understanding of the pathophysiological mechanism of normal-appearing WM injury in SLE.

**Supplementary Information:**

The online version contains supplementary material available at 10.1186/s13075-024-03344-3.

## Introduction

Systemic lupus erythematosus (SLE) represents an inflammatory autoimmune disorder that affects multiple organs and systems. The possible etiology of SLE includes microangiopathy, deposition of immune complexes and production of proinflammatory cytokines. These factors can induce cerebral hypoperfusion, microstructural destruction and abnormal neuron metabolism. Neuropsychiatric systemic lupus erythematosus (NPSLE) is a severe complication of SLE with poor quality of life and high mortality [1]. Neuropsychiatric (NP) syndromes are diverse and nonspecific, ranging from psychiatric symptoms, e.g., headaches and cognitive disorders, to nerve symptoms, e.g., demyelination and epilepsy, making it difficult to attribute clinical NP syndromes to SLE-related mechanisms. However, the lack of criteria for early diagnosis and limited comprehension of its pathogenesis, or the difficulties in attributing NP symptoms to SLE constitute a significant clinical challenge.

SLE patients with no NP syndromes are considered non-NPSLE cases [2]. Although without NP syndromes, several investigators have found brain structural and functional alterations, and abnormalities in normal-appearing white matter (WM) microstructure in non-NPSLE patients. For example, Li et al. found thinner cortical thickness and abnormal topological organization in non-NPSLE patients [3]. Kozora and colleagues found that white matter microstructural integrity is compromised in the absence of cognitive decline by diffusion tensor imaging (DTI) [4]. These findings indicate the brain of non-NPSLE patients has undergone subtle changes before progression to NPSLE. Thus, identifying the changes of white matter microstructure in non-NPSLE patients may potentially benefit early detection of brain injury and enhance our understanding of the pathophysiological mechanisms of SLE [5]. In this study, to detect the early changes in the central nervous systems (CNS) of SLE patients, and to avoid confounding factors of NP-related symptoms and structrual changes, we focused on non-NPSLE patients with normal-appearing WM. This may help enhance our understanding of how SLE affects brain microstructure and detect brain abnormalities before NP symptoms appear.

Current studies of white matter microstructure in SLE are mostly based on a conventional single-shell DTI model that has been broadly used as a research tool in CNS [6, 7]. DTI assumes water molecule diffusion obeys the Gaussian distribution. Actually, diffusion in biological tissues has a non-Gaussian pattern due to complex microenvironments (e.g., cell membranes and complex fiber arrangements), which cannot be well characterized by the DTI model. Thus, advanced diffusion models are required to more accurately represent tissue microstructure.

Diffusion kurtosis imaging (DKI), an extension of the DTI model, quantifies the degree of deviation from Gaussian diffusion by introducing the kurtosis term [8]. Besides diffusion tensor-related metrics including fractional anisotropy (FA), mean diffusivity (MD), axial diffusivity (AD) and radial diffusivity (RD), DKI provides kurtosis tensor-related metrics such as mean kurtosis (MK), axial kurtosis (AK) and radial kurtosis (RK). As a complement to the DTI technique, DKI could provide more detailed information about the complex microstructure and has higher sensitivity to subtle brain changes. So far, research on SLE utilizing DKI had demonstrated its efficacy in detecting microstructural changes in the brain [9]. However, it is worth noting that studies applying DKI to SLE remain relatively scarce. Even so, both DTI and DKI models are based on signal representation to indirectly characterize tissue microstructure without considering the biological characteristics of the tissue and lack specificity.

Neurite orientation dispersion and density imaging (NODDI) is a multi-compartment biophysical model which assumes that the tissue microenvironment in each voxel is composed of three compartments: intra-cellular, extra-cellular and free water. Accordingly, NODDI-derived metrics include neurite density index (NDI), orientation dispersion index (ODI), and isotropic diffusion compartment (VISO). Specifically, NDI represents the intracellular volume fraction that mainly reflects axonal density. ODI represents neurite orientation dispersion that characterizes the coherence of fiber bundle orientation. ODI, ranging from 0 to 1, is high in loosely organized white matter and low in roughly parallel bundles. VISO represents the free water fraction of isotropic components, e.g., cerebrospinal fluid and extracellular tissue edema. As NODDI could provide biological insights into the alterations of WM microstructure, it has been increasingly popular and could serve as a highly sensitive and specific tool for early diagnosis of various brain disorders, including Parkinson’s disease, degenerative brain disorders, mild traumatic brain injury and tuberous sclerosis [10–13]. However, no studies have evaluated brain WM changes in SLE patients using NODDI models. This data gap underscores the need for this study.

Given the advantages of DKI and NODDI over DTI, we hypothesized that these tools can provide more detailed information about pathophysiological mechanism as well as potentially serve as new biomarkers of microstructural alterations of the brain normal-appearing WM in non-NPSLE patients.

This study aimed to (i) investigate the microstructural changes of WM in non-NPSLE patients by DKI and NODDI and (ii) identify the most discriminative metrics and WM regions as biomarkers of non-NPSLE. Tract-based spatial statistics (TBSS) and atlas-based region-of-interest (ROI) analyses were used to examine the roles of DKI and NODDI parameters in the early detection of white matter changes in non-NPSLE patients and to investigate their ability in discriminating patients from healthy individuals. Additionally, correlation analysis was performed to assess the associations of clinical score with DKI and NODDI metrics in white matter tracts.

## Methods

### Subjects

This was a retrospective case-control study, approved by the Nanfang Hospital of Southern Medical University. Patients from March 2014 to January 2015 with SLE were included in this study. All subjects are from China and signed informed consent before enrollment. Patients were diagnosed with SLE using the 1997 ACR SLE classification criteria [14]. Complete neurological and neuropsychological examinations were performed by a rheumatologist (Q. H.) to ensure no nervous system involvement. Inclusion criteria were: age between 15 and 53 years; female gender; right-handedness; no MRI contraindication or brain parenchymal signal-intensity abnormalities on T2/FLAIR sequences; no symptoms of anxiety and depression as determined by the Self-rating Anxiety Scale (SAS) and Self-rating Depression Scale (SDS) for non-NPSLE patients. Exclusion criteria were: a history of psychiatric or neurological diseases, diabetes, thyroid disease, stroke, tumor, head trauma, alcoholism or substance abuse and cardiovascular disease (hypertension, coronary heart disease, hyperlipidemia, cardiomyopathy). Clinical indicators for non-NPSLE patients were recorded, including age, gender, education years, duration of illness, Systemic Lupus International Collaborating Clinics/American College of Rheumatology Damage Index (SLICC/ACR) damage index (SDI) scores [15], Systemic Lupus Erythematosus Disease Activity Index (SLEDAI) score [16], C3, C4 and CH50.

### Image acquisition

All subjects were scanned on a 3.0-T Philips Medical Achieva Systems MR scanner with an 8-channel phased-array head coil at Nanfang Hospital, Southern Medical University, Guangzhou, China. Axial T2WI and FLAIR scans were carried out to rule out participants with visible brain lesions. Multi-shell diffusion MRI data were acquired with a spin-echo (SE) single-shot echo planar imaging (SS-EPI) sequence with the following parameters: TR/TE, 2000/69 ms; acquisition matrix, 88 × 88; field of view, 224 × 224 mm^2^; in-plane resolution, 2.5 × 2.5 mm^2^; slice thickness, 3 mm without gap; 44 axial slices; b = 1,000/2,000 s/mm^2^; 32 diffusion-weighting directions at each b value, and one b = 0 s/mm^2^ scan (b0). Diffusion images with obvious artifact, excessive head movement, serious noise or signal loss were excluded.

### Preprocessing and parameter estimation

Data pre-processing involves the following steps accomplished with the FSL package (https://fsl.fmrib.ox.ac.uk/fsl/fslwiki*)* [17]. Firstly, eddy current and motion correction were performed to correct image misalignment along different diffusion encoding directions and image deformation caused by vortex in the gradient coil. Then, skull-stripping was performed for b0 images in each subject using Brain Extraction Tool in FSL. Then, preprocessed multi-shell dMRI data were employed to assess DKI metrics (FA, MD, AD, RD, MK, AK and RK) with DIPY (https://dipy.org/) [18, 19] and NODDI metrics (NDI, ODI and VISO) with AMICO (https://github.com/daducci/AMICO) [20]. Given that the non-Gaussian components of the diffusion signal are particularly sensitive to noise and artefacts, before conducting DKI fitting for kurtosis metrics, we performed noise and artefacts suppression by using Gaussian smoothing with a Gaussian kernel with fwhm = 2.5 [8].

### Tract-based spatial statistics (TBSS)

TBSS analysis of DKI and NODDI was carried out with the FSL package. FA data were aligned to the MNI (Montreal Neurological Institute) standard space using nonlinear registration. Then, mean FA was generated and thinned to create a skeleton. All DKI and NODDI-related parameter maps were projected onto the skeleton for voxel-wise statistical analysis.

Nonparametric permutation tests (5000 permutations) were performed with the “randomize” function in the FSL toolbox. Then, the family-wise error (FWE) was corrected for multiple comparisons using threshold-free cluster enhancement (TFCE) and significant between-group differences were identified with cluster size larger than 100 voxels [21, 22]. The John Hopkins University (JHU) white-matter tractography atlas [23] was utilized to describe the regions of each significant cluster. For cluster analysis, DKI and NODDI metrics were averaged within each significant cluster. Then, receiver operating characteristic (ROC) curve analysis and area under the curve (AUC) were used to assess the power of each DKI and NODDI metric to discriminate SLE patients from healthy controls.

### Region of interest (ROI) analysis

Twenty WM tracts provided by the JHU white-matter tractography atlas were analyzed as regions of interest. Binary ROI masks were used to extract mean values for NODDI and DKI metric maps, which have been aligned to the MNI standard space in the TBSS procedure. Then, Student’s T-test analysis of each WM tract was performed to determine group differences with false discovery rate (FDR) correction and effect size calculated by Cohen’s d. In addition, ROC curves and AUC values were used to define the discriminative abilities of DKI and NODDI metrics in each WM tract between non-NPSLE patients and healthy controls.

### Correlation analysis of parameters and clinical data

Spearman’s correlation analysis was performed to assess the associations of the mean metric values of significantly different WM tracts based on ROI analysis with disease course, C3, C4, CH50, and SLEDAI score.

## Results

### Study population

A total of 57 non-NPSLE patients and 43 healthy controls were included in this study. After screening, totally 49 female patients with non-NPSLE and 41 age-, sex-, and education-matched healthy controls (HC) were eligible. For non-NPSLE patients, 8 subjects were excluded due to a history of psychiatric or neurological diseases (*n* = 3), a lack of clinical information (*n* = 4) and invalid head movement correction (*n* = 1). For healthy controls, 2 subjects were excluded due to invalid head movement correction. The detailed information of clinical indicators for participants is shown in Table 1 and Supplementary Table S1.

**Table 1:** Clinicodemographic characteristics of the participants

	Non-NPSLEs (*n* = 49)	HCs (*n* = 41)	Z values	*p*
Female	49	41		
Age (years)	29.8 ± 10.3 [15–53]	29.9 ± 9.08 [14–52]	Z = 0.68	0.75
Education (years)	10.1 ± 4.13 [0–18]	12.8 ± 3.98 [0–21]	Z = 1.32	0.06
Disease duration (years)	3.21 ± 4.04 [0.04-17]	\		
SLEDAI	10.4 ± 6.98 [0–23]	\		
SDI (0:1:2:3)	17:23:4:1	\		
C3 (g/l)	0.58 ± 0.30 [0.11–1.25]	\		
C4 (g/l)	0.11 ± 0.08 [0.02–0.29]	\		
CH50 (µ/ml)	31.6 ± 20.6 [0.02–68.1]	\		

Note: The Kolmogorov-Smirnov Z test was used to assess group differences. HC: Healthy Control; non-NPSLE: Non-neuropsychiatric Systemic Lupus Erythematosus; SLEDAI: Systemic Lupus Erythematosus Disease Activity Index; SDI: Systemic Lupus International Collaborating Clinics /American College of Rheumatology (SLICC/ACR) Damage Index; Serology: C3, C4 and CH50. Data are mean ± SD [range]

### TBSS analysis

**Fig. 1:**
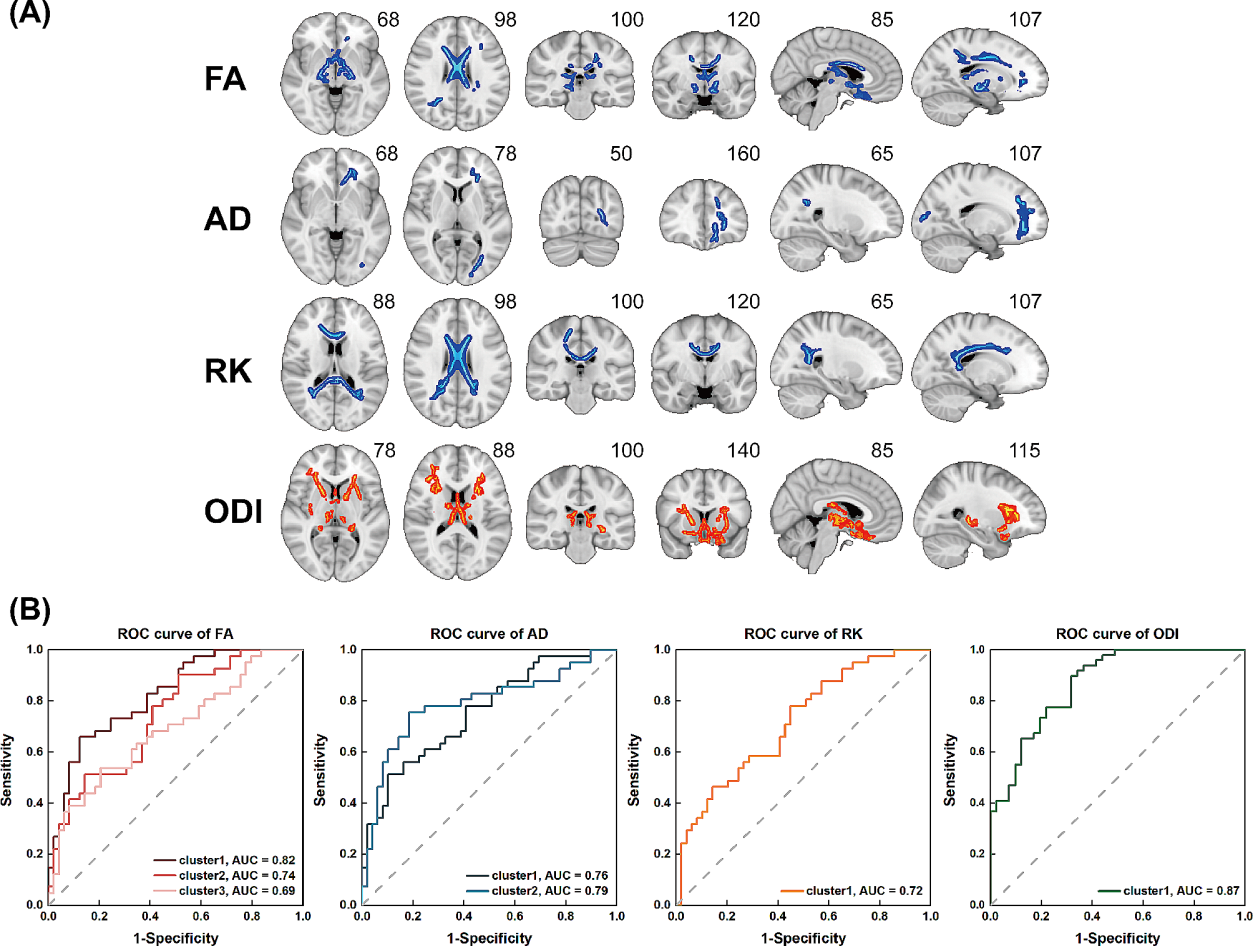
TBSS and ROC curve analyses. (**A**) TBSS analysis showed clusters of significant differences in DKI (FA, AD and RK) and NODDI (ODI) metrics between non-NPSLE patients and the healthy group (*P* < 0.05, FWE corrected). FA: Fractional Anisotropy; AD: Axial Diffusivity; RK: Radial Kurtosis; ODI: Orientation Dispersion Index. Red and blue denote increase and decrease in the non-NPSLE group, respectively. The superscript number in each image refers to its slice. (**B**) Receiver operating characteristic (ROC) curves of averaged FA, AD, RK and ODI values in each significant cluster identified by tract-based spatial statistics analysis (TBSS). AUC, Area under the ROC Curve

Figure 1A; Table 2 show the significant clusters of DKI and NODDI metrics in TBSS analysis. Compared with the healthy group, the non-NPSLE group had significantly decreased FA, AD and RK and markedly increased ODI mostly in the anterior thalamic radiation (ATR), cingulum (cingulate gyrus, CG), inferior fronto-occipital fasciculus (IFOF), superior longitudinal fasciculus (SLF), splenium and genu of corpus callosum (F_major and F_minor) and inferior longitudinal fasciculus (ILF). Besides, FA and RK were significantly decreased in the corticospinal tract (CST) and cingulum (hippocampus, CH) while ODI values were also significantly increased in the uncinate fasciculus (UF) and CST in the patient group. Cluster sizes in TBSS analysis were more than 7000 voxels in FA (7814), RK (7062) and ODI (7542), which were much more than in AD (1817). In addition, ODI had higher statistical significance (*p* = 0.007) compared with FA (*p* = 0.019, 0.022, 0.043), AD (*p* = 0.035, 0.022) and RK (*p* = 0.012). In ROC curve analysis (Fig. 1B), the ODI cluster showed the largest area under the curve (AUC = 0.869), with the optimal cutoff value of 0.223, a sensitivity of 89.8% and a specificity of 68.3%. Detailed information about ROC analysis is provided in Supplementary Table S2.

**Table 2:** White matter tracts in TBSS analysis based on DKI and NODDI

Metrics	Cluster index	Number of voxels	signal peaks (x, y, z)			Minimally *p*-value^#^	White matter tracts
FA	1	4659	15	-34	7	0.019	ATR_L, ATR_R, F_minor, CST_R, CST_L, UF_L, IFOF_L, CG_L, UF_R, IFOF_R
	2	2794	-14	-4	32	0.022	CST_L, F_minor, CG_L, SLF_L, ATR_L, CG_R, ATR_R
	3	361	26	-55	24	0.043	IFOF_R, SLF_R, ILF_R, F_major, CH_R, ATR_R
AD	1	1286	-24	37	-2	0.035	F_minor, IFOF_L, ATR_L, UF_L, CG_L, SLF-T_L, SLF_L
	2	531	-25	-79	11	0.022	F_major, IFOF_L, ILF_L, SLF_L, SLF-T_L, ATR_L
RK	1	7062	12	-1	30	0.012	F_major, F_minor, CST_R, ILF_L, CG_L, IFOF_L, IFOF_R, SLF_L, SLF-T_L, CG_R, SLF_R, ATR_L, ATR_R, ILF_R, CH_R, CH_L, SLF-T_R, CST_L
ODI	1	7542	-9	23	-12	0.007	ATR_R, ATR_L, IFOF_R, IFOF_L, UF_L, UF_R, F_minor, SLF_L, SLF_R, CST_L, ILF_L, SLF-T_L, CG_L, SLF-T_R

#Family-wise error (FWE) corrected at *p* < 0.05. The ranks of white matter tracts follow the probabilities of cluster masks being a member of regions within the JHU white matter tractography atlas. FA: Fractional Anisotropy; AD: Axial Diffusivity; RK: Radial Kurtosis; ODI: Orientation Dispersion Index; L: Left; R: Right; ATR: Anterior thalamic radiation; CST: Corticospinal tract; CG: Cingulate Gyrus; CH: Cingulum Hippocampus; F_major: Forceps Major; F_minor: Forceps Minor; IFOF: Inferior Fronto-occipital Fasciculus; ILF: Inferior Longitudinal Fasciculus; SLF: Superior Longitudinal Fasciculus; SLF-T: Superior Longitudinal Fasciculus (temporal part); UF: Uncinate Fasciculus.

### Atlas-based ROI analysis

**Fig. 2:**
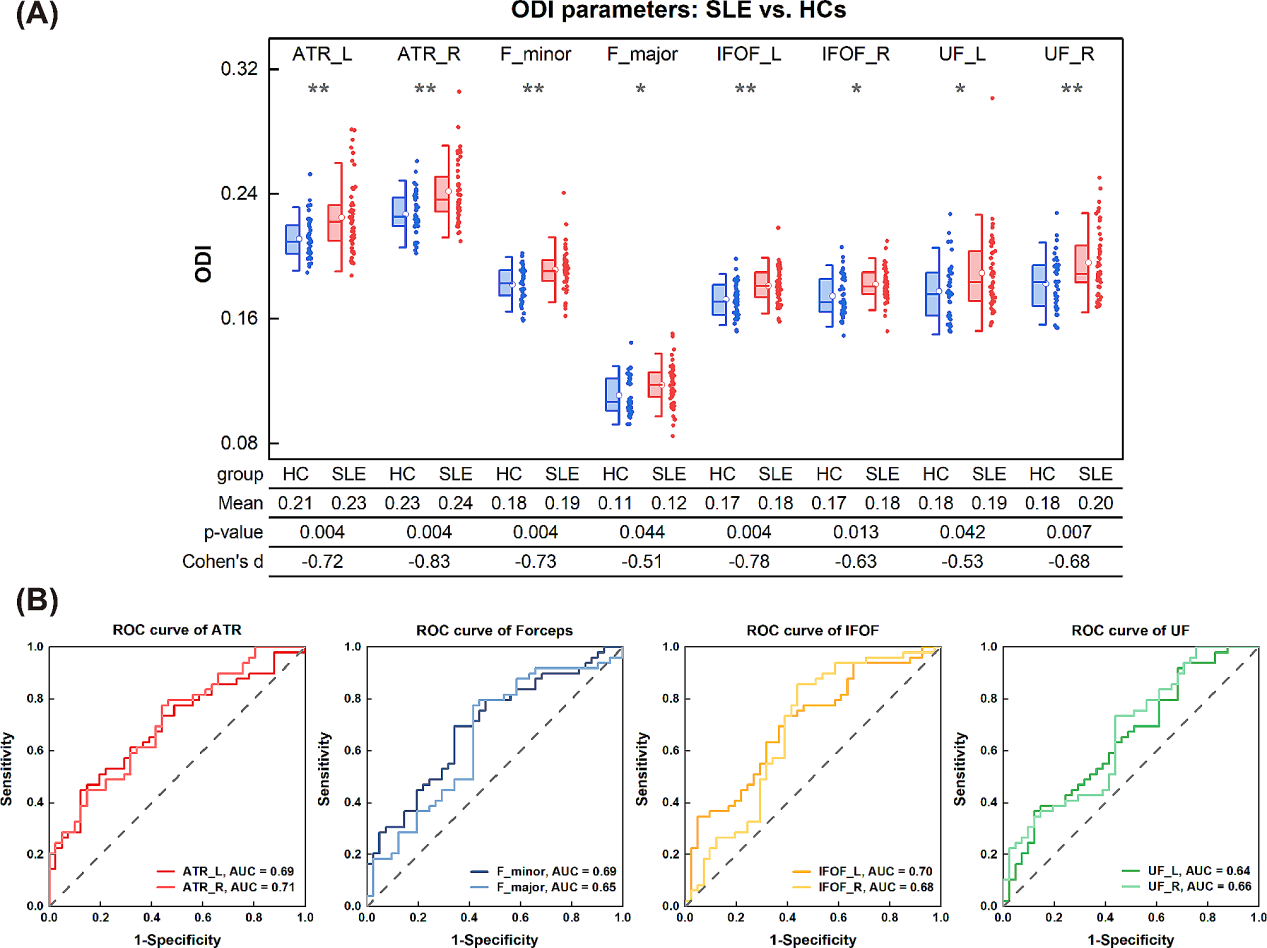
Atlas-based ROI and ROC curve analyses. (**A**) Atlas-based region of interested (ROI) analysis of the non-NPSLE group versus healthy controls. Mean ODI values of left/right significant JHU tracts in the Student’s t test with FDR correction. Scatterplots show the mean ODI values of various JHU tracts for each participant. Boxplots show median, mean, minimum, maximum and interquartile range. *P < 0.05 (two-tailed), **P < 0.01 (two-tailed). L: Left; R: Right; ATR: Anterior Thalamic Radiation; F_minor: Forceps Minor; F_major: Forceps Major; IFOF: Inferior Fronto-occipital Fasciculus; UF: Uncinate Fasciculus. (**B**) Receiver operating characteristic (ROC) curves for averaged orientation dispersion index (ODI) values in significant John Hopkins University (JHU) tracts. AUC: Area under the ROC Curve

The four key metrics (FA, AD, RK and ODI) obtained from voxel-wise TBSS analysis were studied in atlas-based ROI analysis. After FDR correction, only ODI exhibited a significant difference between the patient and healthy control groups, and significant differences were mainly found in ATR, corpus callosum (CC), IFOF and UF (Fig. 2A). The Students’ t test results for these four key metrics (FA, AD, RK and ODI) in each ROI (totally 20 ROIs) with FDR-corrected *p* values and effect sizes (Cohens’ d) are also shown in Supplementary Table S3. In addition, ROC curve analysis is shown in Fig. 2B, and detailed information is listed in Supplementary Table S4. ODI in the right ATR had the largest AUC (AUC = 0.706) in discriminating patients from healthy controls. For the right ATR, the optimal cutoff value was 0.227, with a sensitivity of 77.6% and a specificity of 56.1%.

### Correlation analysis

**Fig. 3:**
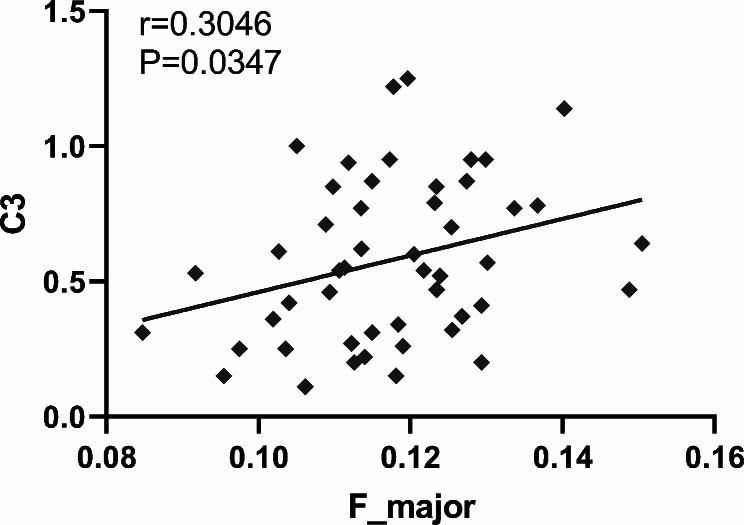
C3 shows a positive correlation with ODI in F_major. Spearman correlation analysis of C3 and F_major of orientation dispersion index (ODI) in the non-NPSLE group. Average ODI in F_major correlated positively with C3 value. F_major: Forceps Major

As shown in Fig. 3, mean ODI in F_major was positively correlated with C3 scores. No correlations were found between ODI values and disease course, SLEDAI, C50 and C4.

## Discussion

To the best of our knowledge, this is the first study using NODDI models to investigate the microstructural changes of the WM in SLE patients. Furthermore, we compare the discriminative abilities of DKI and NODDI metrics within TBSS clusters and each JHU WM region (totally 20 regions). The main findings of this study are as follows: (i) significantly reduced FA, AD and RK and markedly increased ODI were found in multiple WM regions in non-NPSLE patients compared with controls; (ii) ODI in ATR_R had the greatest discriminative power; and (iii) C3 scores were correlated with mean ODI in F_major.

Our data showed lower FA in non-NPSLE patients mainly in the thalamus and frontal and parietal lobes, which basically corroborated previous DTI studies of non-NPSLE [24–27]. Reduced FA might reflect damage to the myelin sheath surrounding axons, reduced axonal packing density or enhanced membrane permeability [28]. In addition, reduced AD was mainly found in left frontal and occipital lobes in this study. Decreased AD indicates axonal injury, reduced axonal caliber, or less coherent orientation of axons [28]. Our results about AD metric*s* are different from findings reported by previous DTI studies on SLE [29, 30]. For example,. Zhao et al. reported non-NPSLE patients had increased AD in bilateral corticospinal tracts and reduced AD in right superior longitudinal fasciculus-temporal terminations [30]. These discrepant results may be explained by the fact that complex cellular components and structures cannot be well described using the DTI model. We also detected reduced radial kurtosis (RK) in patients with non-NPSLE versus healthy controls mainly in the parietal lobe. RK quantifies the limitation of water molecules diffusion, which is affected by cell membranes and myelin [31]. Reduced RK suggests the diffusion of water molecules is closer to the Gaussian distribution and decreased restriction in the diffusion environment [10, 32]. However, no significant differences were observed in RD and AK. We hypothesize that this sensitivity difference may be attributed to specific directional diffusivities and kurtosis. Water molecules exhibit a more uniform diffusion along axonal tracts, resulting in a Gaussian distribution, which can be detected by the axial diffusivity metrics (AD). Conversely, motion is constrained by the complex microstructure in the perpendicular direction, resulting in a non-uniform (non-Gaussian) distribution, and this phenomenon can be detected by radial kurtosis metrics (RK). Therefore, the combined axial diffusivity metrics (AD) and radial kurtosis metrics (RK) can sensitively and accurately reflect changes in the direction of neural fibers. In addition to the DKI results, increased ODI was found in frontal lobe and thalamus, showing a high overlap with the regions of reduced FA while no NDI alterations were detected in this study. Increased ODI indicates the presence of fiber crossing and dispersion [33]. Orientation dispersion index and neurite density are two major aspects of FA [34]. Elevated ODI values within the brain may indicate a change in the morphology of the microstructure rather than in axon density, suggesting that ODI provides more specific information than FA. Therefore, DKI and NODDI metrics were complementary in revealing the underlying mechanism of white matter impairment in SLE. Additionally, the ODI cluster showed the highest statistical significance and the largest AUC in ROC curve analysis, suggesting ODI is more sensitive than DKI metrics. Hence, NODDI could serve as a more sensitive and specific biomarker for the detection of WM abnormalities in non-NPSLE patients that merits further study.

In atlas-based ROI analysis, after FDR correction, only ODI showed a significant difference, indicating that ODI has an advantage in detecting WM fibers over DKI metrics. We found higher ODI values for non-NPSLE patients in bilateral ATR, IFOF, UF and CC (F_major and F_minor). ATR is the predominant component of the anterior limb of the internal capsule (ALIC), which is associated with cognitive functions, including memory encoding and executive function. Mamah et al. revealed asymmetric microstructural changes of the right ALIC in patients with schizophrenia, which correlated with cognitive abnormalities [35]. In addition, our ROC curve analysis showed ODI in the right ATR had the best discriminative ability, indicating that ODI in the right ATR may serve as a potential biomarker of SLE, facilitating early detection and diagnosis. IFOF and UF are association fibers that connect different cortical areas on the same side. IFOF is the main structural pathway for language semantics, and UF is mostly involved in episodic memory, language and socioemotional processing [36]. Comprising commissural fibers, the CC is the largest white matter inter-hemispheric commissure. CC integrity plays a crucial role in sensory-motor functions, attention, language and memory [37]. Previous findings suggested that the morphology of the corpus callosum is associated with several psychiatric disorders, including schizophrenia and bipolar disorder [38]. Abnormalities in ATR, IFOF, UF and CC may account for the manifestation of cognitive dysfunction in SLE patients, including damaged executive function, memory, verbal ability, etc. Future studies combining cognitive and behavioral indicators with fibers in these regions may help explain the NP symptoms of SLE.

In correlation analysis, ODI in F_major was positively correlated with C3 level, as shown above. It was suggested that ODI has a strong correlation with microglial density [39]. The splenium of the corpus callosum (F_major) involves different caliber axonal fibers and the most compact area of glial cells in the CC, affecting language, visual information transfer and behavior, possibly responsible for changes in consciousness. The complement system is involved in the process of neuroinflammation in SLE, and previous studies have demonstrated that low C3 and C4 levels are potential diagnostic markers that could help monitor disease activity in SLE [40]. Additionally, microglia and astrocytes play a protective role in the brain by synthesizing and secreting complement components [41]. Therefore, the detected positive correlation may be because microglial activation in F_major increases the production of complement C3 proteins (higher C3); meanwhile activated microglia reduce the coherence of axonal orientation (higher ODI).

Taken together, TBSS and atlas-based ROI analyses by combining DKI and NODDI metric*s* can provide a more comprehensive understanding of WM alterations in non-NPSLE patients. ROC curve analysis demonstrated that ODI, rather than DKI, is the most sensitive and specific biomarker. These findings indicate that dMRI is beneficial for identifying subclinical brain structural involvement before the onset of NP symptoms. Consequently, it suggests the necessity for earlier clinical intervention to prevent the progression of brain damage. Furthermore, a deeper understanding of the complex mechanisms of the CNS in SLE helps explain the progression of the disease, facilitating early clinical diagnosis and careful consideration of treatment strategies.

The limitations of this study were as follows. First, due to the absence of NPSLE data, differences among NPSLE, non-NPSLE patients and healthy controls could not be examined. Secondly, we focused solely on the changes of brain WM microstructure in SLE. Future studies should evaluate the gray matter based on the multi-shell dMRI technique. Finally, external validation data were not available to assess the reproducibility and generalizability of the current results.

In summary, microstructural changes in brain WM in non-NPSLE were assessed by analyzing the properties of different biological tissues with the DKI and NODDI models. TBSS analysis revealed reduced FA, AD and RK and increased ODI in multiple WM regions in non-NPSLE patients, which may be attributed to demyelination in myelinated axons and the morphological changes of fibers. Atlas-based ROI analysis reported increased ODI values in 4 fibers, and the right ATR had the highest ability to distinguish non-NPSLE from healthy controls, indicating that ODI in the right ATR may serve as a potential biomarker of SLE. The positive correlation between C3 and ODI in F_major may be associated with microglial activation following WM’s microstructural changes. Our findings provide new insights into CNS microstructural injury and its potential mechanisms in SLE patients, even in the absence of NP symptoms. This contributes to early diagnosis and emphasizes the necessity of early clinical intervention for SLE patients.

### Electronic supplementary material

Below is the link to the electronic supplementary material.

Supplementary Material 1

## Data Availability

No datasets were generated or analysed during the current study.

## Abbreviations

SLE: Systemic lupus erythematosus
NPSLE: Neuropsychiatric systemic lupus erythematosus
non-NPSLE: Systemic lupus erythematosus patients without neuropsychiatric symptoms
DTI: Diffusion tensor imaging
DKI: Diffusion kurtosis imaging
NODDI: Neurite orientation dispersion and density imaging
WM: White matter
FA: Fractional anisotropy
MD: Mean diffusivity
AD: Axial diffusivity
RD: Radial diffusivity
MK: Mean kurtosis
AK: Axial kurtosis
RK: Radial kurtosis
NDI: Neurite density index
ODI: Orientation dispersion index
VISO: Volume fraction of the isotropic diffusion compartment
TBSS: Tract-based spatial statistics
ROI: Region-of-interest
JHU: John Hopkins University
