# Supplementary material for: Microstructural changes of the white matter in systemic lupus erythematosus patients without neuropsychiatric symptoms: a multi-shell diffusion imaging study
Source: Arthritis Res Ther. 2024 May 28;26:110. doi: 10.1186/s13075-024-03344-3 (PMC11134659; doi:10.1186/s13075-024-03344-3)
Supplement: Supplementary file 1 — Supplementary Material 1 [file 13075_2024_3344_MOESM1_ESM.doc]

**Supplementary material**

**Supplementary Table S1: Clinical manifestations, serology and treatment of non-NPSLE patients.**

|  | Non-NPSLEs (n=49) |
| --- | --- |
| ACR criteria, n(%) |  |
| Malar rash | 10 (20) |
| Discoid lupus | 17 (35) |
| Photosensitivity | 0 (0) |
| Aphthosis | 1 (2) |
| Arthritis | 19 (39) |
| Serositis | 5 (10) |
| Lupus nephritis | 34 (69) |
| Neurological disorder | 0 (0) |
| Haematological disorder | 42 (86) |
| Immunological disorder | 49 (100) |
| Antinuclear antibodies | 49 (100) |
| Serology, mean (S.D.) |  |
| ANA (IU/ml) | 228.92 (158.54) |
| Anti-dsDNA (IU/ml) | 168.42 (152) |
| Anti-Sm (IU/ml) | 47.76 (73.95) |
| Treatment, n(%) |  |
| Glucocorticoid | 49 (100) |
| Immunosuppressants | 19 (39) |

ACR: American College of Rheumatology; ANA:Antinuclear antibody; Anti-Sm: anti-Smith antibodies; Anti-dsDNA: anti-double stranded DNA.

**Supplementary Table S2.** **Discrimination results of each clusters of diffusion metrics**

| Metrics | Cluster index | Optimal cutoff | Sensitivity | Specificity | AUC | SE | 95% CI | |
| --- | --- | --- | --- | --- | --- | --- | --- | --- |
|  |  |  |  |  |  |  | Lower | Upper |
| FA | 1 | 0.419 | 0.659 | 0.878 | 0.822 | 0.0431 | 0.738 | 0.907 |
|  | 2 | 0.597 | 0.902 | 0.490 | 0.742 | 0.0514 | 0.641 | 0.842 |
|  | 3 | 0.458 | 0.537 | 0.796 | 0.691 | 0.0561 | 0.581 | 0.801 |
| AD | 1 | 0.001 | 0.512 | 0.898 | 0.756 | 0.0505 | 0.657 | 0.855 |
|  | 2 | 0.002 | 0.756 | 0.816 | 0.789 | 0.0508 | 0.689 | 0.889 |
| RK | 1 | 1.311 | 0.780 | 0.551 | 0.719 | 0.0533 | 0.614 | 0.823 |
| ODI | 1 | 0.223 | 0.898 | 0.683 | 0.869 | 0.0367 | 0.797 | 0.941 |

Custer index represents the voxel intensity. AUC: areas under the receiver operating characteristic curve; SE: Standard Error; CI: Confidence Interval; FA: Fractional Anisotropy; AD: Axial Diffusivity; RK: Radial Kurtosis, ODI: Orientation Dispersion Index.

**Supplementary Table S3 The Student’s T-test analysis results of diffusion metrics in each JHU tracts with FDR correction**

| Index | Tract | SLEs | HCs | p | Cohen's d | SE Cohen's d |
| --- | --- | --- | --- | --- | --- | --- |
| FA |  |  |  |  |  |  |
| 1 | ATR_L | 0.396±0.016 | 0.404±0.015 | 0.16025 | 0.486 | 0.218 |
| 2 | ATR_R | 0.377±0.015 | 0.385±0.013 | 0.16025 | 0.566 | 0.221 |
| 3 | CG_L | 0.525±0.027 | 0.533±0.020 | 0.35980 | 0.323 | 0.215 |
| 4 | CG_R | 0.452±0.027 | 0.456±0.032 | 0.76535 | 0.132 | 0.212 |
| 5 | CH_L | 0.388±0.027 | 0.386±0.025 | 0.81682 | -0.072 | 0.212 |
| 6 | CH_R | 0.388±0.027 | 0.386±0.025 | 0.81682 | -0.072 | 0.212 |
| 7 | CST_L | 0.555±0.018 | 0.558±0.017 | 0.67227 | 0.178 | 0.213 |
| 8 | CST_R | 0.553±0.019 | 0.555±0.017 | 0.81682 | 0.083 | 0.212 |
| 9 | F_minor | 0.438±0.016 | 0.444±0.014 | 0.33740 | 0.351 | 0.215 |
| 10 | F_major | 0.567±0.022 | 0.577±0.016 | 0.16025 | 0.494 | 0.219 |
| 11 | IFOF_L | 0.446±0.019 | 0.453±0.014 | 0.20362 | 0.419 | 0.217 |
| 12 | IFOF_R | 0.440±0.018 | 0.444±0.015 | 0.58772 | 0.238 | 0.213 |
| 13 | ILF_L | 0.437±0.019 | 0.439±0.016 | 0.76535 | 0.119 | 0.212 |
| 14 | ILF_R | 0.453±0.022 | 0.454±0.017 | 0.85227 | 0.051 | 0.212 |
| 15 | SLF-T_L | 0.494±0.033 | 0.493±0.033 | 0.93307 | -0.018 | 0.212 |
| 16 | SLF-T_R | 0.531±0.030 | 0.544±0.026 | 0.16790 | 0.457 | 0.218 |
| 17 | SLF_L | 0.388±0.014 | 0.392±0.015 | 0.35980 | 0.312 | 0.214 |
| 18 | SLF_R | 0.391±0.017 | 0.394±0.016 | 0.67227 | 0.205 | 0.213 |
| 19 | UF_L | 0.409±0.023 | 0.412±0.023 | 0.76535 | 0.129 | 0.212 |
| 20 | UF_R | 0.404±0.020 | 0.408±0.019 | 0.67227 | 0.184 | 0.213 |
| AD [μm^2^/ms] |  |  |  |  |  |  |
| 1 | ATR_L | 1.371±0.086 | 1.337±0.057 | 0.38602 | -0.456 | 0.218 |
| 2 | ATR_R | 1.345±0.070 | 1.323±0.042 | 0.38602 | -0.368 | 0.216 |
| 3 | CG_L | 1.449±0.043 | 1.471±0.062 | 0.38602 | 0.42 | 0.217 |
| 4 | CG_R | 1.352±0.047 | 1.367±0.062 | 0.49731 | 0.259 | 0.214 |
| 5 | CH_L | 1.297±0.043 | 1.300±0.041 | 0.90748 | 0.077 | 0.212 |
| 6 | CH_R | 1.300±0.073 | 1.278±0.041 | 0.38602 | -0.359 | 0.215 |
| 7 | CST_L | 1.443±0.029 | 1.444±0.044 | 0.90748 | 0.037 | 0.212 |
| 8 | CST_R | 1.490±0.030 | 1.491±0.032 | 0.94363 | 0.015 | 0.212 |
| 9 | F_minor | 1.496±0.053 | 1.498±0.050 | 0.90748 | 0.04 | 0.212 |
| 10 | F_major | 1.685±0.090 | 1.712±0.067 | 0.38602 | 0.326 | 0.215 |
| 11 | IFOF_L | 1.384±0.034 | 1.396±0.037 | 0.38602 | 0.352 | 0.215 |
| 12 | IFOF_R | 1.391±0.036 | 1.397±0.031 | 0.70928 | 0.179 | 0.213 |
| 13 | ILF_L | 1.374±0.036 | 1.379±0.052 | 0.80122 | 0.131 | 0.212 |
| 14 | ILF_R | 1.392±0.039 | 1.393±0.033 | 0.90748 | 0.037 | 0.212 |
| 15 | SLF-T_L | 1.445±0.057 | 1.441±0.067 | 0.90748 | -0.064 | 0.212 |
| 16 | SLF-T_R | 1.529±0.051 | 1.545±0.050 | 0.38602 | 0.319 | 0.215 |
| 17 | SLF_L | 1.261±0.033 | 1.251±0.043 | 0.49731 | -0.273 | 0.214 |
| 18 | SLF_R | 1.293±0.034 | 1.287±0.027 | 0.64150 | -0.211 | 0.213 |
| 19 | UF_L | 1.347±0.058 | 1.340±0.046 | 0.80122 | -0.124 | 0.212 |
| 20 | UF_R | 1.374±0.058 | 1.365±0.045 | 0.70928 | -0.169 | 0.212 |
| RK |  |  |  |  |  |  |
| 1 | ATR_L | 1.108±0.039 | 1.126±0.043 | 0.41296 | 0.438 | 0.217 |
| 2 | ATR_R | 1.107±0.045 | 1.123±0.043 | 0.51720 | 0.36 | 0.215 |
| 3 | CG_L | 1.111±0.041 | 1.123±0.049 | 0.52228 | 0.284 | 0.214 |
| 4 | CG_R | 1.066±0.043 | 1.066±0.050 | 0.98077 | 0.013 | 0.212 |
| 5 | CH_L | 0.921±0.029 | 0.915±0.032 | 0.71369 | -0.182 | 0.213 |
| 6 | CH_R | 0.919±0.033 | 0.917±0.035 | 0.97374 | -0.068 | 0.212 |
| 7 | CST_L | 1.430±0.057 | 1.450±0.067 | 0.51720 | 0.324 | 0.215 |
| 8 | CST_R | 1.451±0.060 | 1.473±0.067 | 0.51720 | 0.332 | 0.215 |
| 9 | F_minor | 1.079±0.053 | 1.085±0.042 | 0.95752 | 0.117 | 0.212 |
| 10 | F_major | 1.285±0.070 | 1.318±0.067 | 0.41296 | 0.478 | 0.218 |
| 11 | IFOF_L | 1.095±0.046 | 1.108±0.040 | 0.52228 | 0.294 | 0.214 |
| 12 | IFOF_R | 1.112±0.045 | 1.121±0.037 | 0.67065 | 0.22 | 0.213 |
| 13 | ILF_L | 1.073±0.039 | 1.069±0.043 | 0.95752 | -0.09 | 0.212 |
| 14 | ILF_R | 1.131±0.043 | 1.126±0.046 | 0.95752 | -0.103 | 0.212 |
| 15 | SLF-T_L | 1.181±0.058 | 1.193±0.067 | 0.71369 | 0.188 | 0.213 |
| 16 | SLF-T_R | 1.283±0.068 | 1.300±0.066 | 0.58630 | 0.253 | 0.213 |
| 17 | SLF_L | 1.145±0.047 | 1.147±0.054 | 0.98077 | 0.036 | 0.212 |
| 18 | SLF_R | 1.221±0.045 | 1.221±0.056 | 0.98077 | 0.008 | 0.212 |
| 19 | UF_L | 0.929±0.043 | 0.929±0.041 | 0.98077 | -0.005 | 0.212 |
| 20 | UF_R | 0.936±0.046 | 0.939±0.043 | 0.97374 | 0.06 | 0.212 |
| ODI |  |  |  |  |  |  |
| 1 | ATR_L | 0.225±0.023 | 0.211±0.013 | **0.00430** | -0.718 | 0.226 |
| 2 | ATR_R | 0.242±0.019 | 0.227±0.014 | **0.00362** | -0.828 | 0.231 |
| 3 | CG_L | 0.138±0.017 | 0.131±0.014 | 0.06154 | -0.474 | 0.218 |
| 4 | CG_R | 0.178±0.023 | 0.177±0.027 | 0.91975 | -0.046 | 0.212 |
| 5 | CH_L | 0.146±0.025 | 0.147±0.017 | 0.99182 | 0.002 | 0.212 |
| 6 | CH_R | 0.163±0.027 | 0.157±0.023 | 0.51784 | -0.234 | 0.213 |
| 7 | CST_L | 0.134±0.011 | 0.136±0.010 | 0.66125 | 0.145 | 0.212 |
| 8 | CST_R | 0.137±0.010 | 0.139±0.010 | 0.51784 | 0.216 | 0.213 |
| 9 | F_minor | 0.191±0.014 | 0.182±0.012 | **0.00430** | -0.73 | 0.227 |
| 10 | F_major | 0.118±0.013 | 0.111±0.012 | **0.04354** | -0.513 | 0.219 |
| 11 | IFOF_L | 0.181±0.012 | 0.172±0.011 | **0.00424** | -0.775 | 0.228 |
| 12 | IFOF_R | 0.182±0.011 | 0.175±0.013 | **0.01303** | -0.627 | 0.223 |
| 13 | ILF_L | 0.182±0.015 | 0.180±0.013 | 0.66125 | -0.145 | 0.212 |
| 14 | ILF_R | 0.176±0.019 | 0.174±0.017 | 0.71076 | -0.121 | 0.212 |
| 15 | SLF-T_L | 0.129±0.026 | 0.128±0.024 | 0.93017 | -0.031 | 0.212 |
| 16 | SLF-T_R | 0.116±0.016 | 0.113±0.017 | 0.52731 | -0.202 | 0.213 |
| 17 | SLF_L | 0.236±0.013 | 0.233±0.010 | 0.51784 | -0.227 | 0.213 |
| 18 | SLF_R | 0.244±0.017 | 0.242±0.016 | 0.74018 | -0.103 | 0.212 |
| 19 | UF_L | 0.189±0.025 | 0.178±0.018 | **0.04170** | -0.527 | 0.22 |
| 20 | UF_R | 0.196±0.021 | 0.183±0.017 | **0.00688** | -0.684 | 0.225 |

Note: p<0.05 were in bold. SE: Standard Error; FA: Fractional Anisotropy; AD: Axial Diffusivity; RK: Radial Kurtosis; ODI: Orientation Dispersion Index; L: Left; R: Right; ATR: Anterior thalamic radiation; CST: Corticospinal tract; CG: Cingulate Gyrus; CH: Cingulum Hippocampus; F_major: Forceps Major; F_minor: Forceps Minor; IFOF: Inferior Fronto-occipital Fasciculus; ILF: Inferior Longitudinal Fasciculus; SLF: Superior Longitudinal Fasciculus; SLF-T: Superior Longitudinal Fasciculus (temporal part); UF: Uncinate Fasciculus.

**Supplementary Table S4. Discrimination results of significant JHU fibers**

| Tract | Optimal cutoff | Sensitivity | Specificity | AUC | SE | 95% CI | |
| --- | --- | --- | --- | --- | --- | --- | --- |
|  |  |  |  |  |  | Lower | Upper |
| ATR_L | 0.225 | 0.449 | 0.878 | 0.691 | 0.0565 | 0.581 | 0.802 |
| ATR_R | 0.227 | 0.776 | 0.561 | 0.706 | 0.0542 | 0.600 | 0.813 |
| F_minor | 0.188 | 0.694 | 0.659 | 0.693 | 0.0558 | 0.583 | 0.802 |
| F_major | 0.109 | 0.776 | 0.585 | 0.653 | 0.0585 | 0.538 | 0.768 |
| IFOF_L | 0.175 | 0.735 | 0.610 | 0.701 | 0.0552 | 0.593 | 0.809 |
| IFOF_R | 0.172 | 0.857 | 0.561 | 0.680 | 0.0570 | 0.568 | 0.792 |
| UF_L | 0.194 | 0.367 | 0.878 | 0.643 | 0.0580 | 0.529 | 0.757 |
| UF_R | 0.184 | 0.735 | 0.561 | 0.662 | 0.0570 | 0.550 | 0.773 |

AUC: areas under the receiver operating characteristic curve; SE: Standard Error; CI: Confidence Interval; L: Left, R:Right, ATR: Anterior Thalamic Radiation, F_minor: Forceps Minor, F_major: Forceps Major, IFOF: Inferior Fronto-occipital Fasciculus, UF: Uncinate Fasciculus.
